# Exploring Variable Approaches in Complex Hernia Repair: A Comprehensive Literature Review

**DOI:** 10.7759/cureus.60181

**Published:** 2024-05-13

**Authors:** Javier Meza-Hernandez, Paulina Elizabeth Huchim-Servín, Andrea Escamilla-Lopez, David Villanueva-Lechuga

**Affiliations:** 1 Faculty of Medicine, National Autonomous University of Mexico, Mexico City, MEX

**Keywords:** botulinum toxin type a, tissue expanders, mesh fixation, surgical techniques, complex hernia

## Abstract

Surgeons have long grappled with categorizing complex hernias, leading to varied interpretations and fluctuating incidence rates. Complex Abdominal Wall Reconstruction (CAWR) addresses repairs for large hernias, with defined factors including size, previous repairs, mesh placement, infections, and comorbidities. This review explores pivotal surgical techniques for complex hernia repair, starting with Preoperative Progressive Pneumoperitoneum (PPP) and progressing to innovative methods like Botulinum Toxin Type A. Mesh fixation, both open and laparoscopic, plays a crucial role, with synthetic and biological mesh options discussed. Hybrid techniques and the "sandwich" approach are proposed for intricate cases. Each technique presents advantages and limitations, emphasizing the ongoing quest for optimal outcomes.

## Introduction and background

An abdominal wall hernia consists of a protrusion of intra-abdominal tissue through a fascial defect in the abdominal wall [1]. Over the years, surgeons have grappled with categorizing complex hernias and identifying them as challenges demanding technical expertise and extended surgical durations. The absence of a clear definition has led to varied interpretations among authors, resulting in a fluctuating incidence of complex hernias. As outlined by the European Collaborative for Abdominal Wall Reconstruction, complex hernias encompass negative factors such as size exceeding 10 cm, previous repairs, prior mesh placement, infections, and comorbidities. Complex Abdominal Wall Reconstruction (CAWR) addresses repairs for large hernias and involves procedures such as component separation, adhesiolysis, or flap reconstruction [2]. Data from the Danish Ventral Hernia Database indicate that 15% of all repairs target patients with incisional hernias > 15 cm [3]. In a systematic review by Deerenberg, large hernias (>10 cm) were classified as 'simple' in 80% and as 'complex' (involving tissue loss, intra-abdominal infection, infected mesh, or parastomal hernia repair) in 20% of cases. The most recent definition is abdominal wall hernia complex if it exhibits at least one of the following factors: width exceeding 10 cm, parastomal hernia, infected mesh, presence of a stoma, fistula or abscess, or loss of domain greater than 20% [4]. Patients with at least one modifiable risk factor, such as a body mass index (BMI) > 30 kg/m^2^, active nicotine use, diabetes mellitus (HbA1c > 6.5), chronic obstructive pulmonary disease (COPD) (beyond Gold I), use of immunosuppressive medication, or a metabolic equivalent (MET) score < 4, fall into this category. Throughout this evolutionary process, a myriad of surgical techniques have emerged to address these types of hernias. In the following sections, we will delve into the most important ones.

## Review

**Figure 1:**
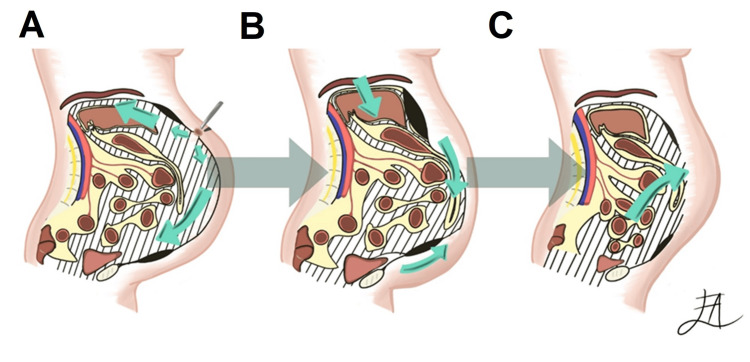
Preoperative progressive pneumoperitoneum A. Carbon dioxide is gradually and controllably introduced into the peritoneal cavity, creating a space within the abdomen. B. As air fills the peritoneal cavity, the viscera shifts to fill the space created by the pneumoperitoneum. C. After surgery the viscera tend to revert to their original position due to air pressure and patient repositioning. However, the abdominal tissue, including skin and muscles, stays stretched due to the pneumoperitoneum. Image credits: Andrea Escamilla-López

The first technique, described in 1947 by Ivan Goñi Moreno, is the Preoperative Progressive Pneumoperitoneum (PPP) technique. It involves acting as a pneumatic tissue expander to gradually stretch the abdominal muscles and increase abdominal volume, facilitating closure of the abdominal wall without tension. This results in respiratory benefits such as stabilizing respiratory capacity, enhancing diaphragmatic tone, and improving the efficiency of other respiratory muscles. This method consists of gradually stretching the abdominal muscles by insufflating air into the abdominal cavity at regular intervals, either through puncturing the abdominal wall or using an intraabdominal catheter. The catheter is ideally positioned away from hernias and previous incisions; the most common place where the puncture site is made is the left hypochondrium or Palmer’s point to avoid the liver and spleen [5]. Approximately 15 to 20 liters of air are insufflated over a period of three to six weeks to stretch the abdominal wall and adapt organ systems [6] (Figure 1).

The primary indication for this technique is in hernias with a loss of domain, characterized by the presence of a large visceral sac that has evolved over many years and cannot be reduced into the abdominal cavity. Other indications include ventral hernias exceeding 10 cm in size, sizable inguinal and umbilical hernias with irreducible sacs, and loss of domain hernias with an estimated volume exceeding 10 liters. It is also recommended for substantial recurrent hernias associated with infected meshes that require mesh removal. [7]. While this technique can be applied to these types of hernias, there is a debate about what exactly constitutes the primary indication for this technique. In 62.1% of cases, it is at the discretion of the surgeon without consideration of imaging studies. In 11.3% of cases, surgeons rely on volume criteria from computed tomography, such as a Tanaka index greater than 20%. Finally, in 20.7% of cases, a combination of criteria is used, such as a Tanaka index greater than 25% and a transverse diameter of the hernia greater than 10 cm, or a large incisional hernia with a transverse diameter defect greater than 10 cm [2].

Success in reducing hernia size depends on several factors, with the most critical being the ratio between hernia volume (HV) and abdominal volume (PV). This means that, if the ratio between hernia volume and abdominal volume decreases by less than 20% following PPP treatment, there is a strong likelihood (89%) that the patient can undergo successful surgical repair with primary fascial closure [8].

The complications associated with the PPP procedure are a crucial aspect to consider in clinical practice. Among the most common complications are abdominal and shoulder pain, as well as subcutaneous emphysema. Although these clinical manifestations are frequent, they typically do not require intervention and can be managed with analgesics and careful observation. However, in severe cases, delaying the next insufflation or adjusting the administered gas volume may be necessary. Another reported complication is dyspnea, especially relevant in patients with chronic respiratory diseases. While it is generally manageable with observation, modifying the insufflation protocol may be necessary in severe cases. Furthermore, more serious complications, such as pneumothorax, require a conservative approach with active observation. Deep vein thrombosis (DVT) and pulmonary thromboembolism (PTE) are potentially serious complications that can be prevented with appropriate antithrombotic measures, such as subcutaneous heparin administration and the use of compression stockings on the lower extremities. Infections and issues associated with special insertion devices, such as dialysis catheters and reservoir ports, have also been documented. These cases often require surgical correction and, in some instances, antibiotic treatment while continuing with the PPP [2].

**Figure 2:**
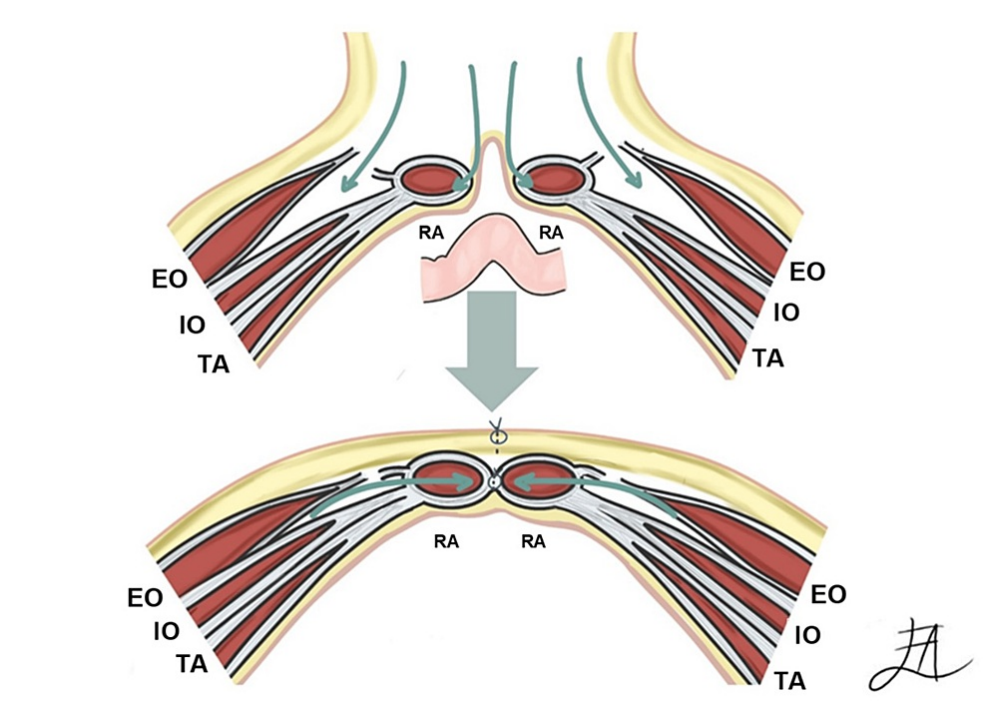
Component separation technique A. Separation of the external oblique muscle from the rectus abdominis muscle along the linea semiluniaris is performed. B. This separation allows for medial advancement of the rectus abdominis-internal oblique myofascial complex, reducing tension and facilitating primary repair. EO: External Oblique, IO: Internal Oblique, TA: Transversus Abdominis, RA: Rectus Abdominis Image credits: Andrea Escamilla-López.

The next technique is component separation, developed in 1990 by Ramirez et al. [9]. It is employed for the reconstruction of the abdominal wall, particularly after preoperative techniques such as PPP or Botulinum Toxin Type A treatments. The main indications are complex ventral hernias accompanied by significant defects in the abdominal wall. It involves strategically advancing the musculofascial tissue, comprising muscles and layers covering the hernia, to effectively cover the defect and fortify the abdominal wall. The dissection process occurs either unilaterally or bilaterally in the external/internal oblique plane, tailored to the unique characteristics of the hernia and patient. The skin and subcutaneous fat are lifted from the fascia of the rectus muscle and the external oblique muscle and its fascia to prepare the repair area. A fasciotomy of the external oblique aponeurosis is performed, meaning the fibrous layer covering the external oblique muscle is cut. Subsequently, the areolar tissue plane between the internal and external oblique muscles is dissected to separate them and allow medial advancement of the rectus muscle. Once the rectus muscles are approximated in the midline, the fascia is closed with sutures, with the length of sutures being at least four times the length of the wound. A polypropylene (PPL) or polyvinylidene (PVDF) mesh is placed "onlay" (over the fascia) and secured with non-absorbable staples or sutures between the internal and external muscles. Crucially, the preservation of deeper neurovascular structures is prioritized to prevent potential damage and complications [10] (Figure 2).

Despite its merits, this technique has limitations in terms of musculofascial advancement across various regions of the abdominal wall. Notably, the maximum advancement achievable was 20 cm at the waistline, 10 cm at the epigastrium, and 6 cm at the suprapubic region. This specificity underscores the nuanced nature of this procedure. Furthermore, the reported hernia recurrence rate was up to 30%, with no clear-cut variables directly linked to recurrence. Another consideration is that tension-free closure, a desired outcome, may not be consistently attained even in hernias smaller than those initially studied. These nuances highlight the evolving landscape of hernia repair techniques and the ongoing quest for optimal outcomes to address these challenging medical conditions [7,9,11].

In 1997, Jacobsen et al. introduced a novel approach to address complex hernias using tissue expanders for massive and persistent abdominal hernias [12]. This technique entails the strategic placement of expanders beneath the external oblique fascia in the abdominal wall, allowing for the restoration of anatomical relationships without causing harm to the muscles or neurovascular structures, ultimately achieving tension-free closure. Over a period of four to eight weeks, the inserted expanders were gradually filled. Prior to hernia closure, expanders are drained, and a precise definition of hernia edges is established. Subsequently, the skin grafts applied to the abdominal viscera were excised, and the skin was closed over the fascial repair. It is important to note that this method has some disadvantages, including the potential for expander rupture, hernia recurrence, and complications during the removal of skin grafts such as enterotomy. It's worth mentioning that much of the evidence supporting this technique is derived from case series [12,13]. A systematic review conducted in 2021 analyzed 7767 patients who underwent various tissue expander procedures, including 150 cases of abdominal wall reconstruction [14]. The review revealed that tissue expanders were primarily used in 82% of ventral hernia repair cases and 18% in congenital defects like omphalocele. These expanders were typically placed in different planes: subcutaneous (50%), intermuscular (26.7%), intra-abdominal (10.0%), and in multiple planes or unspecified locations (13.3%). The most commonly reported complications included tissue expander failure (5.3%), infection (4.7%), exposure (2.7%), hematoma (7.3%), wound dehiscence (2.7%), superficial infection (3.3%), and skin flap necrosis (0.67%). Additionally, other complications, such as enterocutaneous fistula, insufficient skin for closure, femoral nerve neuropraxia, port failure, and small bowel gangrene, were documented. The recurrence of ventral hernia repair is between 8.1%and 11.7% [15]. In another review [15], four articles were analyzed regarding the utilization of the tissue expander technique in the presence of a stoma. The reason is that stoma introduces challenges, as it may potentially act as a source of contamination and hinder the feasibility of utilizing a component separation technique due to its location. In Carr's study [16], the presence of a stoma did not increase the risk of tissue expander (TE) infection in three patients with hernias resulting from trauma and prior abdominal surgery. Carr suggested that strategic incision placement away from the stoma and minimizing the duration between the two stages could help mitigate complications. In contrast, the review [15] says that pediatric transplant patients experienced implant-related complications necessitating surgical intervention, particularly among those with stomas and severe medical conditions. Additionally, infectious complications were observed in stomas during Stage I, although the direct correlation remained unclear. These findings imply an elevated risk of expander-related complications in patients with comorbidities, particularly chronic illness and immunosuppression, when a stoma is present. Despite these challenges, meticulous consideration and management of the stoma are crucial for all patients undergoing tissue expander techniques. To optimize outcomes in tissue expander surgery, various modifications are proposed. Firstly, employing small, laterally positioned skin incisions facilitates optimal placement of the tissue expander (TE) [16]. Placing the TE within non-scarred, undisturbed soft tissues is also crucial to promote successful integration [12]. For optimal results, intermuscular placement between the external and internal oblique muscles is preferred, as it offers an avascular plane and minimizes the risk of neurovascular damage [12]. Commencing expansion, no sooner than two weeks after the insertion allows for sufficient healing at the incision site, while a gradual expansion over four weeks to three months, tailored to the patient's comfort and compliance, is recommended [12]. Aiming for a 10%-20% greater expansion than the implant's capacity is advised to accommodate tissue growth adequately [17]. Additionally, in cases of skin-grafted ventral hernias, allowing a maturation period of 3-12 months before definitive repair is prudent [18]. Synthetic mesh should be avoided in instances of contamination or high infection risk [19]. During fascial closure, vigilant monitoring of airway pressure, particularly in patients with extensive loss of domain, is essential [12]. In cases where airway pressures exceed 30-40 cm H_2_O or 5-10 cm H_2_O over baseline, incomplete closure is recommended to prevent abdominal compartment syndrome [12]. Postoperatively, continuous monitoring for signs of abdominal compartment syndrome is imperative. If there is insufficient skin during Stage II closure, options such as leaving some skin graft intact, promoting wound closure by secondary intention, or replacing and re-expanding tissue expanders may be considered [12].

Mesh fixation techniques play a crucial role in minimizing recurrence rates, and are considered the gold standard for addressing larger defects. The choice of fixation technique significantly affects factors such as recurrence rates, chronic pain, health-related quality of life (HRQOL), and complication rates. Two primary surgical approaches are discussed: open surgery, involving the closure of abdominal wall layers and the potential placement of additional mesh to prevent recurrence, and laparoscopic surgery, which offers precise visualization of the hernia defect, adhesion lysis, and more accurate mesh application, with a recurrence rate of 4-9%. In terms of mesh type, they fall into two categories: synthetic and biological. Synthetic meshes can be either absorbable or non-absorbable. Light meshes are recommended for common hernias and tension-free primary closures. Absorbable synthetic meshes, such as vicryl and dexon, find utility as patches for hernia sac segments when peritoneal closure proves challenging. On the other hand, biological meshes, including pigskin and human skin and intestine, become viable options when issues like infection, bacterial contamination, or inflammatory responses to foreign bodies hinder the use of synthetic prostheses. Hybrid techniques, which combine aspects of both laparoscopic and open procedures, have been proposed as innovative approaches for ventral hernia repair. These techniques aim to leverage the benefits of each method, providing a comprehensive solution to enhance surgical outcomes [20]. For patients necessitating intricate abdominal wall reconstructions, especially those with thin abdominal walls, denervation, and a heightened risk of recurrence due to associated comorbidities, the "sandwich" or double mesh technique emerges as a promising solution. This procedure involves the meticulous resection of the previous surgical scar and a thorough dissection through planes surrounding the hernia defect until identifying the aponeurosis for incision. Throughout this process, preserving the hernia sac is a paramount consideration. Following this, the midline is once again approximated using the component separation technique levels I and II. This intricate step entails bilaterally detaching the external oblique and the posterior rectus aponeurosis to dissect the retro-muscular space, culminating in the placement of a robust polypropylene mesh. Subsequently, the midline is closed, and a lightweight polypropylene mesh is strategically positioned supra-aponeurotically, secured with 0-caliber non-absorbable monofilament sutures placed transfascially. In instances where adequate access to the retro-muscular space poses challenges, the intraperitoneal onlay mesh (IPOM) with tissue-separating mesh proves to be a valuable alternative. Here, the hernia defect is meticulously approximated, and a pre-aponeurotic heavy polypropylene mesh is thoughtfully situated. In all cases, the incorporation of two closed drains with active suction in the subcutaneous tissue is a systematic practice. This technique has demonstrated remarkable efficacy and reproducibility, particularly in patients dealing with giant hernias, multiple recurrent hernia defects, and comorbidities that elevate the risk of relapses. Importantly, it maintains an acceptable complication rate, underscoring its reliability in addressing complex cases [21].

**Figure 3:**
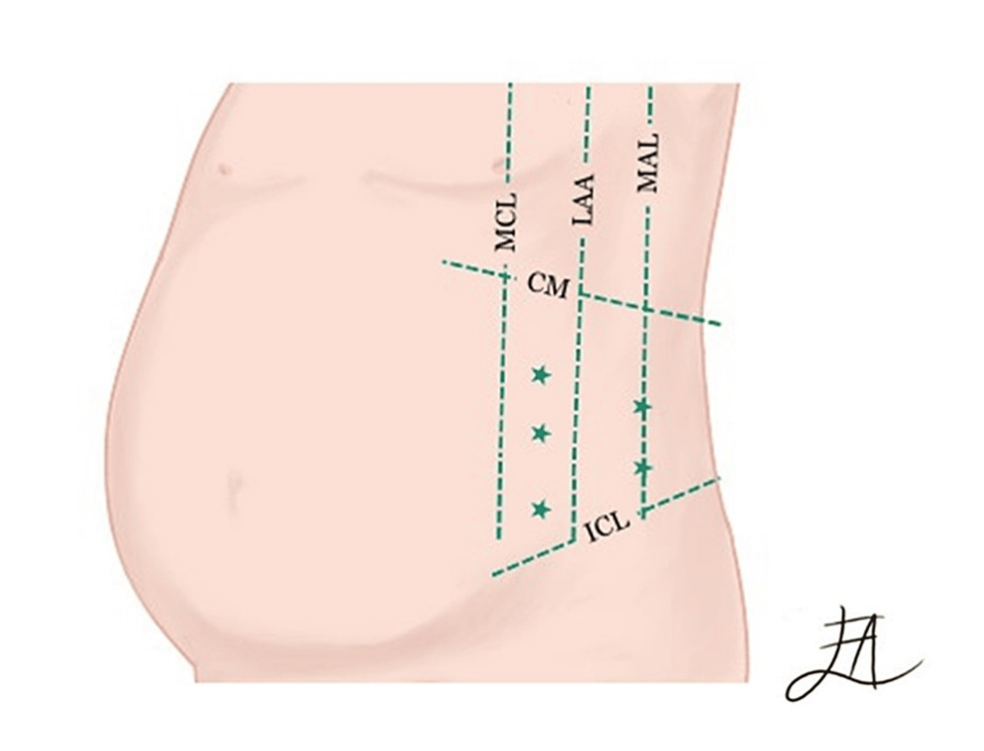
Botulinum Toxin Type A abdominal reconstruction Botulinum Toxin Type A (BTA) injection targets the abdominal wall muscles on the left flank. MAL middle axillary line, AAL anterior axillary line, MCL middle clavicular line, CM costal margin, ICL iliac crest level. Image credits: Andrea Escamilla-López

The most recent technique was first described in 2009, which consists of the use of Botulinum Toxin Type A (BTA) for abdominal reconstruction [22]. This method involves the application of BTA for abdominal reconstruction. By injecting the neurotoxin into muscle tissue, a transient state of flaccid muscle paralysis is induced (Figure 3).

This results in an average extension of 3.2 cm in muscle length on each side, accompanied by a bilateral reduction of 1 cm in muscle thickness. Additionally, there is a notable decrease of 3.5 cm in hernia width [23]. Firstly, a computed tomography (CT) imaging study is conducted, focusing on the hernia defect's size, diameter, and location, as well as the characteristics of the ventral muscle blocks and the depth of the oblique muscles. Following this examination, 100 units of botulinum toxin A, reconstituted with 2 ml of sterile solution, are injected. The injections are strategically administered at 5 points on each side of the aponeurotic defect, totaling 10 units at each point. These injection sites are chosen based on the locations of the highest myoelectric stimulation in the abdominal wall, specifically 2 points in the mid-axillary line between the costal margin and the external iliac crest, and 3 points on the edge of the external oblique muscle [22]. The injection is performed on an outpatient basis and under sterile conditions by the surgical team, guided by tomographic findings regarding depth to infiltrate the internal oblique muscles. The maximum duration of this effect is six months [24]. Nevertheless, the approach to technique, dosage, and timing of placement varies across different studies. Following a four-week interval, patients undergo reevaluation, with new CT scans conducted to gauge aponeurotic advancement before scheduling the intervention. The surgical procedure entails excising the existing scar, meticulously dissecting the sac and hernia ring. The wall plasty is executed with adaptability based on intraoperative findings, consistently prioritizing aponeurotic apposition, and incorporating a polypropylene mesh (either simple or composite). The downside of this technique lies in its limited supporting evidence, as its efficacy is primarily derived from a small number of case studies [24].

## Conclusions

This review navigates the complexity of hernia repair, outlining evolving definitions and exploring various surgical techniques. From Preoperative Progressive Pneumoperitoneum to mesh fixation and innovative approaches like Botulinum Toxin Type A, each method presents distinct advantages and limitations. While not exhaustive, this concise revision captures the essence of the most important techniques in the evolving landscape of complex hernia repair, providing valuable insights for surgeons. To determine the optimal technique, further investigations are crucial. Comparative studies assessing the efficacy, recurrence rates, and patient outcomes of each method, along with considerations for specific hernia characteristics and comorbidities, are necessary. A comprehensive understanding of the evolving landscape of hernia repair techniques will guide surgeons in choosing the most effective approach tailored to individual cases.

## References

[1] Mulita F, Parchas N, Solou K, Tchabashvili L, Gatomati F, Iliopoulos F, Maroulis I (2020). Postoperative pain scores after open inguinal hernia repair: comparison of three postoperative analgesic regimens. Med Arch.

[2] Martínez-Hoed J, Bonafe-Diana S, Bueno-Lledó J (2021). A systematic review of the use of progressive preoperative pneumoperitoneum since its inception. Hernia.

[3] Wegdam JA, de Jong DL, Gielen MJ, Nienhuijs SW, Füsers AF, Bouvy ND, de Vries Reilingh TS (2023). Impact of a multidisciplinary team discussion on planned ICU admissions after complex abdominal wall reconstruction. Hernia.

[4] de Jong DL, Wegdam JA, Berkvens EB, Nienhuijs SW, de Vries Reilingh TS (2023). The influence of a multidisciplinary team meeting and prehabilitation on complex abdominal wall hernia repair outcomes. Hernia.

[5] Elstner KE, Read JW, Rodriguez-Acevedo O, Ho-Shon K, Magnussen J, Ibrahim N (2017). Preoperative progressive pneumoperitoneum complementing chemical component relaxation in complex ventral hernia repair. Surg Endosc.

[6] Van Geffen HJ, Simmermacher RK (2005). Incisional hernia repair: abdominoplasty, tissue expansion, and methods of augmentation. World J Surg.

[7] Bittner R, Bingener-Casey J, Dietz U (2014). Guidelines for laparoscopic treatment of ventral and incisional abdominal wall hernias (International Endohernia Society [IEHS])-Part III. Surg Endosc.

[8] Sabbagh C, Dumont F, Fuks D, Yzet T, Verhaeghe P, Regimbeau JM (2012). Progressive preoperative pneumoperitoneum preparation (the Goni Moreno protocol) prior to large incisional hernia surgery: volumetric, respiratory and clinical impacts. A prospective study. Hernia.

[9] Ramirez OM, Ruas E, Dellon AL (1990). "Components separation" method for closure of abdominal-wall defects: an anatomic and clinical study. Plast Reconstr Surg.

[10] Bueno-Lledó J, Torregrosa A, Ballester N (2017). Preoperative progressive pneumoperitoneum and botulinum toxin type A in patients with large incisional hernia. Hernia.

[11] Franklin BR, Patel KM, Nahabedian MY, Baldassari LE, Cohen EI, Bhanot P (2013). Predicting abdominal closure after component separation for complex ventral hernias: maximizing the use of preoperative computed tomography. Ann Plast Surg.

[12] Jacobsen WM, Petty PM, Bite U, Johnson CH (1997). Massive abdominal-wall hernia reconstruction with expanded external/internal oblique and transversalis musculofascia. Plast Reconstr Surg.

[13] Maroney J, Taylor GA, Lo A, Golpanian S, Prus NW, Livelsberger J, Gassman AA (2020). Ultrasound-guided hydro-dissection facilitates tissue expander placement and components separation in complex ventral hernia repair. Plast Reconstr Surg Glob Open.

[14] Langdell HC, Taskindoust M, Levites HA (2021). Systematic review of tissue expansion: utilization in non-breast applications. Plast Reconstr Surg Glob Open.

[15] Wooten KE, Ozturk CN, Ozturk C, Laub P, Aronoff N, Gurunluoglu R (2017). Role of tissue expansion in abdominal wall reconstruction: A systematic evidence-based review. J Plast Reconstr Aesthet Surg.

[16] Carr JA (2014). Tissue expander-assisted ventral hernia repair for the skin-grafted damage control abdomen. World J Surg.

[17] Alhan D, Şahin İ, Güzey S (2015). Staged repair of severe open abdomens due to high-energy gunshot injuries with early vacuum pack and delayed tissue expansion and dual-sided meshes. Ulus Travma Acil Cerrahi Derg.

[18] Tran NV, Petty PM, Bite U, Clay RP, Johnson CH, Arnold PG (2003). Tissue expansion-assisted closure of massive ventral hernias. J Am Coll Surg.

[19] Rodriguez ED, Bluebond-Langner R, Silverman RP, Bochicchio G, Yao A, Manson PN, Scalea T (2007). Abdominal wall reconstruction following severe loss of domain: the R Adams Cowley Shock Trauma Center algorithm. Plast Reconstr Surg.

[20] Mathes T, Prediger B, Walgenbach M, Siegel R (2021). Mesh fixation techniques in primary ventral or incisional hernia repair. Cochrane Database Syst Rev.

[21] Alpuche HV (2020). "Sandwich" or double mesh technique for the repair of large hernia defects in the elderly: a review of a series of cases (Article in Spanish). Cir gen.

[22] Ibarra-Hurtado TR, Nuño-Guzmán CM, Echeagaray-Herrera JE, Robles-Vélez E, de Jesús González-Jaime J (2009). Use of botulinum toxin type a before abdominal wall hernia reconstruction. World J Surg.

[23] Timmer AS, Claessen JJ, Atema JJ, Rutten MV, Hompes R, Boermeester MA (2021). A systematic review and meta-analysis of technical aspects and clinical outcomes of botulinum toxin prior to abdominal wall reconstruction. Hernia.

[24] Chávez-Tostado KV, Cárdenas-Lailson LE, Pérez-Trigos H (2014). Resultado de la aplicación preoperatoria de toxina botulínica A en el tratamiento de hernias incisionales gigantes. Revista Hispanoamericana de Hernia.

